# Utility of the Cortical Thickness of the Distal Radius as a Predictor of Distal-Radius Bone Density

**DOI:** 10.5812/atr.10687

**Published:** 2013-06-01

**Authors:** Sascha Rausch, Kajetan Klos, Florian Gras, Hristo Kostov Skulev, Albrecht Popp, Gunther Olaf Hofmann, Thomas Mückley

**Affiliations:** 1 Department of Traumatology, Hand and Reconstructive Surgery, Friedrich-Schiller-Universität Jena, Jena, Germany; 2 Arbeitsgemeinschaft für Osteosynthesefragen, Bern, Switzerland; 3 Technical University of Varna, Varna, Bulgaria; 4 Department of Traumatology and Orthopaedic Surgery Helios Klinikum Erfurt, Berlin, Germany

**Keywords:** Distal Radius, Bone Density, Cortical Thickness, Bone Fractures

## Abstract

**Background:**

Bone density is an important factor in the management of fractures of the distal radius.

**Objectives:**

The aim of this study was to establish whether standard anteroposterior (AP) radiographs would provide the attending physician with a prediction of bone density.

**Patients and Methods:**

Six pairs of human cadaveric radii were harvested. The mean donor age was 74 years. Standardized AP radiographs were taken of the radii. The outside diameter and the inside diameter of the cortical shell at the metaphyseal / diaphyseal junction were measured and their ratio was calculated. Dual-energy x-ray absorptiometry (DXA) was used to obtain the bone mineral density (BMD) of the distal parts of the radii. The correlation of the BMD values with these ratios was studied.

**Results:**

The mean BMD was 0.559 (SD = 0.236) g / cm^2^. The mean outside diameter/inside diameter ratio was 1.24 (SD = 0.013); the ratio significantly correlated with the total BMD (P = 0.001; R^2^ = 0.710). In the BMD subregions, the correlation was also significant.

**Conclusions:**

The outside diameter/inside diameter ratio at the metaphyseal/diaphyseal junction of the distal radius on AP radiographs is suitable for use as a predictor of distal-radius bone density. Further studies should be performed, and clinical utility evaluated.

## 1. Background

Fractures of the distal radius are encountered mainly in postmenopausal women, but may occur in any subject with osteoporosis (1). A variety of management options have been devised. Since the outcome of treatment appears to depend to a large extent, on bone density (2, 3), this factor should be taken into account when choosing the most suitable management strategy for each patient. However, distal radius fractures are very common and frequently occur when full imaging facilities are not available. As a result, the medical staff on duty will rarely be able to determine bone quality as a guide for treatment planning. In the absence of more sophisticated techniques, many physicians will look at standard radiographs and intuitively use the cortical thickness of the distal radius as a rough indicator of bone density. This approach was first described, for the femoral diaphysis, by Barnett and Nordin (4). Similarly, the cortical thickness of the humerus has been used to estimate osteoporosis (5, 6).

## 2. Objectives

The present study was performed with the objective of finding a simple approach for the assessment of bone density in the distal radius, for round-the-clock use by the emergency unit traumatologist or the operating surgeon. The technique was intended to be independent from more sophisticated facilities, and to provide approximate but reliable information.

## 3. Patients and Methods

### 3.1. Specimen Preparation

A total of 12 human cadaveric radii (6 pairs) were harvested from four female and two male donors. The mean donor age was 74 years (64 to 82). The specimens were obtained from voluntary human donors that had agreed to the use of their body for education and research after death and had died of illnesses other than bone diseases. The specimens were de-fleshed, vacuum-packed, and stored at –22°C. Prior to testing, they were thawed at room temperature for 24 hours.

### 3.2. BMD Determination in the Distal Radius

**Figure 1.**
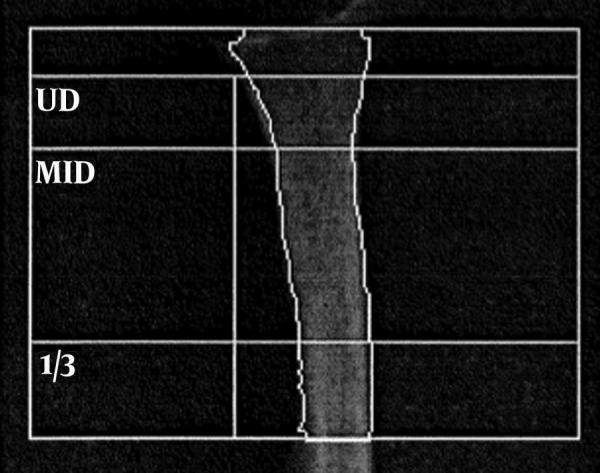
Diagram of the Region of Interest for the DXA Measurement of the BMD Abbreviations: 1/3, one-third distal radius; MID, mid-distal radius; UD, ultra-distal radius

BMD in the distal radius was measured with dual-energy x-ray absorptiometry (DXA). All the BMD measurements were performed by the same investigator (A.P.). The thawed vacuum-packed specimens were placed in a basin filled with a semolina soft-tissue surrogate and positioned horizontally in a DXA machine (Hologic 4500A ^TM^; Hologic, Bedford, MA, USA). Spine phantom scans were performed on a daily basis for quality control. The manufacturer's software was used for analysing the region of interest in the distal radius. The sites chosen were the ultra-distal radius (UD), the mid-distal radius (MID), and the one-third distal radius (1/3) (Figure 1). Each specimen was scanned twice, and the results were averaged. Correlations with the measured cortical thicknesses were calculated for the total BMD, for which the sum of the bone mineral contents (BMCs; g) of the areas was divided by the sum of the areas (cm^2^); and for the different subregions as shown in Figure 1.

### 3.3. Radiography of the Distal Radius

An emergency unit radiographer working with a digital radiography system and employing routinely used techniques and standards performed Anteroposterior (AP) radiographs of the distal radius.

### 3.4. Determination of Cortical Thickness

**Figure 2.**
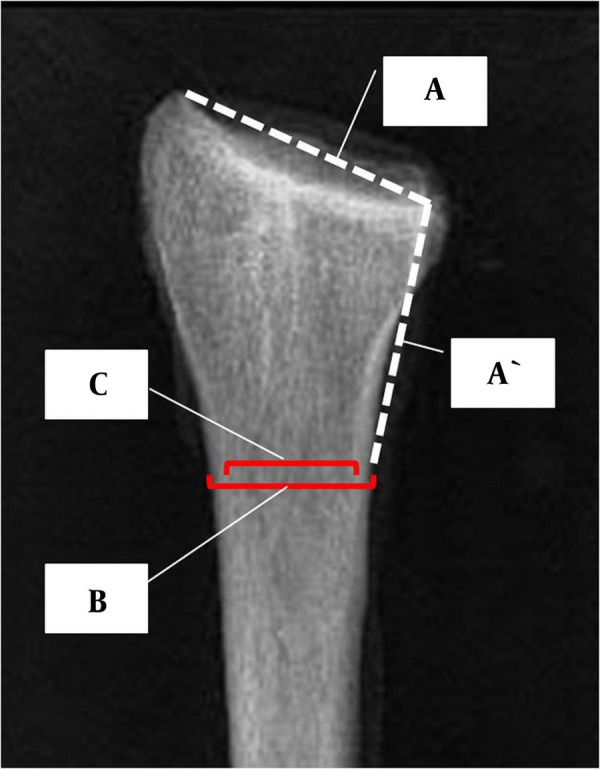
Principle of Establishing the Cortical-Thickness Measuring Site A, line across the radio-carpal joint surface; A', line of same length as A plotted along the radius in a proximal direction; B, line joining the outside (periosteal) borders of the cortical shell of the radius; C, line joining the inside (endosteal) borders of the cortical shell of the radius.

Two independent traumatologists determined the thickness of the distal-radius cortices (S.R. and K.K.). Measurements were performed on digitized radiographs, with the use of Ortho-Tool Cedara 1 Report 5.2 P14 software (Cedara Software Corporation, Milwaukee, WI, USA). First, the measuring site was determined: A line (marked A in Figure 2) was drawn across the radiocarpal joint surface, from the styloid process to the distal radio-ulnar joint, and its length was obtained. Starting at the distal radio-ulnar joint, a line measuring the same length (marked A' in Figure 2) was then plotted in a proximal direction, along the medial border of the radial diaphysis. At the proximal end of this line, the outside diameter of the shaft (between the periosteal cortical borders – marked B in Figure 2) was measured at right angles to the axis of the diaphysis. The inside diameter of the shaft (between the endosteal cortical borders – marked C in Figure 2) was measured at the same site. The B/C ratio was obtained by dividing the outside diameter by the inside diameter.

### 3.5. Statistical Analysis

Inter-observer variability between Observer No. 1 and Observer No. 2 was determined with GLM repeated measures (with Greenhouse-Geisser correction), with BMD as a covariate. Since no significant difference was found between the measurements performed by the two observers, mean values were calculated. The data were normally distributed; therefore, Pearson's correlation test was used. Significance was set at P < 0.05.

## 4. Results

**Figure 3:**
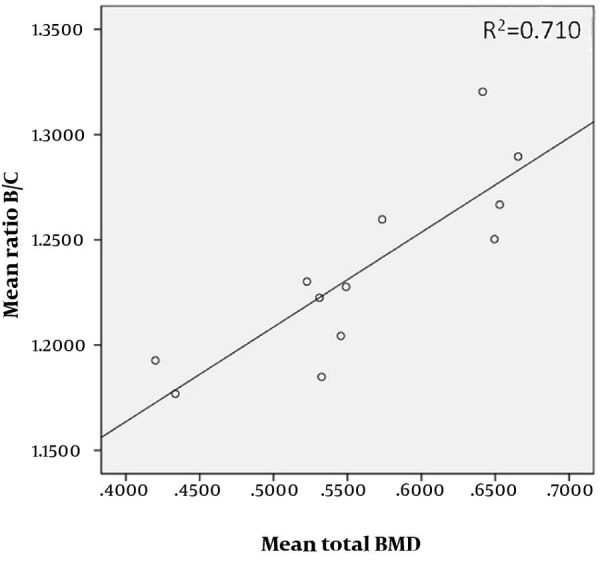
Correlation of the Mean (Observer No. 1 and Observer No. 2 Combined) B/C Ratios and the Mean Total BMD

The detailed results are listed in  Table 1. The measurements obtained by Observer No. 1 did not differ significantly from those obtained by Observer No. 2 (P = 0.997 with total BMD as a covariate; P = 0.751 with UD BMD as a covariate; P = 0.778 with MID BMD as a covariate; P = 0.990 with 1/3 BMD as a covariate). The mean of the B/C ratios found by the two observers correlated significantly with the total BMD (P = 0.001; R^2^ = 0.710) (Figure 3), and with the regional densities (UD BMD: P = 0.002, R^2^ = 0.633; MID BMD: P < 0.001, R^2^ = 0.730; 1/3 BMD: P = 0.004, R^2^ = 0.581).

**Table 1.** Details of the Gender, Age, Length, Total BMD, and Regional BMDs of Each Specimen; and Details of the Distance B, the Distance C, and the B/C Ratio Found by Each Observer, as Well as the Mean B/C Ratios

Serial No.and Side	1 Left	1 Right	2 Right	2 Left	3 Right	3 Left	4 Left	4 Right	5 Left	5 Right	6 Left	6 Right	Mean ± SD
**Radii**													
Gender	Female		Female		Female		Male		Male		Female		
Age, y	82		77		80		64		75		67		74.2
Length, mm	225	225	220	220	225	225	245	245	240	240	230	230	230.8
**BMD ^a^, g /cm^2^**													
Total	0.52	0.53	0.55	0.53	0.57	0.55	0.65	0.67	0.65	0.64	0.43	0.42	0.56 ± 0.08
UD^a^	0.36	0.39	0.38	0.37	0.41	0.41	0.55	0.53	0.54	0.50	0.34	0.34	0.43 ± 0.08
MID^a^	0.55	0.56	0.56	0.56	0.61	0.59	0.64	0.67	0.67	0.67	0.45	0.42	0.58 ± 0.08
1/3^a^	0.64	0.67	0.72	0.71	0.74	0.66	0.82	0.87	0.76	0.79	0.53	0.50	0.70 ± 0.10
**Observer No. 1, mm**													
Distance B	16.3	17.2	17.7	17.6	18.3	18.2	20.1	20.4	18.2	17.2	18.6	18.2	18.17
Distance C	13.2	13.9	14.8	14.4	15.0	14.9	15.5	15.8	14.1	13.5	15.6	15.4	14.68
B/C ratio	1.23	1.24	1.20	1.22	1.22	1.22	1.30	1.29	1.29	1.27	1.19	1.18	1.24 ± 0.04
**Observer No. 2, mm**													
Distance B	16.3	18.0	17.4	19.5	18.5	18.1	23.8	20.5	21.4	17.2	18.0	18.7	18.94
Distance C	13.3	14.9	14.4	17.0	14.2	14.6	19.7	15.9	17.2	12.6	15.5	15.5	15.41
B/C ratio	1.23	1.21	1.21	1.15	1.30	1.23	1.20	1.29	1.24	1.37	1.16	1.20	1.23 ± 0.06
Mean B/C ratio, Observer 1 & 2	1.23	1.22	1.20	1.19	1.26	1.23	1.25	1.29	1.27	1.32	1.18	1.19	1.24 ± 0.04

^a^Abbreviations: 1/3, one-third distal radius; BMD, bone mineral density; MID, mid-distal radius; UD, ultra-distal radius

## 5. Discussion

Indirect radiological techniques such as DXA currently represent the gold standard for the assessment of bone density. However, they are rarely available to the traumatologists at the time of decision-making with regard to the management of the trauma case. This is why efforts are being made to provide the surgeon with direct means of quantifying bone density (7-9). The outcome of the treatment of distal-radius fractures is greatly affected by the quality of the patient's bone stock (2). However, these fractures are common, and treatment decisions should be taken as soon as possible after the traumatic event, which means that routine bone density assessment by the means of techniques such as DXA would be unrealistic. Unlike these more sophisticated methods, radiographs constitute the simplest, standardized and virtually always available diagnostic aid at the early stage of fracture management. The present study was not performed with a view to supersede such methods as DXA and peripheral quantitative computed tomography (pQCT); rather the object was to establish whether an intuitive look at the cortices of the distal radius would allow valid conclusions to be drawn as to the patient's forearm bone density. The results of our study suggest that distal-radius cortical thickness can indeed be used as a predictor of bone quality. Our findings agree with those of Tingart et al. (6), who reported an even stronger correlation between the cortical thickness of the proximal diaphysis of the humerus and the bone quality of the humeral head. The authors also used the metaphysis as the region of interest for BMD determination, with boundaries between sub-regions drawn according to anatomical landmarks of the individual humeri. Cortical thickness was measured at the level of the humeral diaphysis where the endosteal borders of the medial and lateral cortices were parallel to each other. We felt that measurements at that level would be too error-prone in routine emergency-unit practices, and chose to use more obvious landmarks which also take account of the individual patient's anatomy and can be readily seen even in a fractured radius. We decided to use the width of the radio-carpal joint surface, since we think that this dimension can be established with sufficient accuracy even in a Type C fracture pattern. The distal radio-ulnar joint was used as a landmark, since, in fractures of the distal radius, the ulnar is often intact and can provide a useful reference. In the inter-observer comparison, there were no significant differences. Both observers mastered the technique after a short initial training, which confirms the intuitive nature of the method. In summary, we conclude that a visual assessment of the metaphyseal cortices on standard radiographs can provide estimation of distal-radius bone density and in the general trauma context, can be a valid decision-making aid to guide the management of fracture patients. Further studies, including clinical investigations, will be required to evaluate the utility of the technique.
